# Comparison of Fever-reducing Effects in Self-reported Data from the Mobile App: Antipyretic Drugs in Pediatric Patients

**DOI:** 10.1038/s41598-020-60193-1

**Published:** 2020-03-03

**Authors:** Jiyun Choi, Seyun Chang, Jong Gyun Ahn

**Affiliations:** 1 Yonsei University College of Medicine, Seoul, Republic of Korea; 2 Pohang University of Science and Technology, Pohang, Republic of Korea; 3 Mobile Doctor Co., Ltd, Seoul, Republic of Korea; 4 Department of Pediatrics, Severance Children’s Hospital, Yonsei University College of Medicine, Seoul, Republic of Korea

**Keywords:** Paediatric research, Population screening, Information storage

## Abstract

We compared the fever-reducing efficacy of acetaminophen (AA), ibuprofen (IBU), and dexibuprofen (DEX) using data collected from the mobile healthcare application FeverCoach, which provides parents with guidelines for determining their child’s health condition, according to body temperature. Its dataset includes 4.4 million body temperature measurement records and 1.6 million antipyretics treatment records. Changes in body temperature over time were compared after taking one of three different antipyretics (AA, IBU, and DEX), using a one-way ANOVA followed by a post-hoc analysis. A multivariate linear model was used to further analyze the average body temperature differences, calibrating for the influences of age, weight, and sex. Children administered IBU had average body temperatures that were 0.18 °C (0.17–0.19 °C), 0.25 °C (0.24–0.26 °C), and 0.18 °C (0.17–0.20 °C) lower than those of children administered AA, at time intervals of 1–2 hours, 2–3 hours, and 3–4 hours, respectively. Similarly, children administered DEX had average body temperatures that were 0.24 °C (0.24–0.25 °C), 0.28 °C (0.27–0.29 °C), and 0.12 °C (0.10–0.13 °C) lower than those of children administered AA, at time intervals of 1–2, 2–3, and 3–4 hours, respectively. Although the data were collected from the application by non-professional parents, the analysis showed that IBU and DEX were more effective in reducing body temperature than AA was.

## Introduction

Fever is an abnormal increase in body temperature caused by elevation of the hypothalamic set-point^1^. Infectious agents, microbial products, or both as well as cytokines and other inflammatory processes induce macrophages, endothelial cells, and the reticuloendothelial system to synthesize and release pyrogenic cytokines such as interleukin (IL)-1 and IL-6, tumor necrosis factor (TNF)-α, and interferon (IFN)-β and IFN-γ^2^. These cytokines cause the synthesis of prostaglandin E2 (PGE2), which binds to prostaglandin receptors in the hypothalamus to raise the thermostatic set point to febrile levels.

Whether fever is beneficial or harmful is controversial. Potential benefits of fever include inhibition of growth and reproduction of some bacteria and viruses and improvement of immune function at moderately elevated temperatures^3,4^. In older individuals with community-acquired pneumonia, the mortality rate was significantly higher in patients who lacked fever (29%) than in those with fever (4%)^5^. In a study assessing effects of prophylactic antipyretic use during pediatric vaccination, infants administered paracetamol prophylactically at the start of vaccination had lower levels of circulating serotype-specific pneumococcal anticapsular immunoglobulin G for five serotypes (3, 4, 5, 6B, and 23 F) than the controls did^6^.

Although fever plays a role in defending the host from infection, in some situations, it can be more harmful than good^7,8^. Fever increases cardiac output, oxygen consumption, carbon dioxide production, and can exacerbate cardiac insufficiency in patients with cardiovascular and pulmonary systems. Therefore, for a child in shock or with underlying medical conditions that could be exacerbated by increased metabolic demand, fluid, or electrolyte imbalance, appropriate control of the body temperature is essential.

Antipyretics are commonly used to control body temperature. Acetaminophen (AA), the first antipyretic used in modern medicine, and ibuprofen have been the most widely used antipyretics, However the more recently developed dexibuprofen (DEX) series has become common in recent years. AA is recommended at doses of 10–15 mg/kg and should be administered orally once every 4 hours. The antipyretic effects of AA has an onset of between 30 and 60 minutes after administration and normally last for 4 hours^9^. Side effects of AA include liver damage, dizziness, loss of appetite, and skin rash^10^.

IBU, discovered in the 1960s, is a propionic acid derivative and a member of the antipyretic class of nonsteroidal anti-inflammatory drugs (NSAID)^11^. IBU is also effective for pain relief and is often used when pain accompanies inflammation^12^. The optimal dose of IBU is 8–10 mg/kg, which should be administered every 6 hours. The antipyretic effect of IBU commences between 30 minutes and 60 minutes after administration and its main adverse effects are gastrointestinal disorders; allergic reactions affecting the face, tongue, neck, and lips; dyspnea; and urticaria^13^.

The recently developed DEX is a formulation of only the S (+) isomer of IBU. The R (−) IBU has no reducing effects, and while R (−) IBU converts to S(+) IBU, the conversion of R (−) ibuprofen is not immediate^14^. The suggested mode of administration of DEX is not very different from that of IBU, except that the optimal DEX dose is 5–7 mg/kg.

Many studies have investigated the efficacy of various antipyretics^12,14–21^. Although fever and pain are both nonspecific symptoms, pain usually has a direct effect on a patient’s discomfort. Since antipyretics are also used as analgesics, studies of the efficacy of antipyretic drugs have mainly compared their analgesic effects, and relatively few have compare their antipyretic effects.

Several clinical studies have compared antipyretic effects according to drug type^15–17,19–22^. These studies usually used a similar research design with experimental groups divided into several categories and a single drug administration method for every group^15,19–22^. After a specific treatment period, the difference in body temperature reduction was then compared among groups. Usually, the primary endpoint was associated with patient discomfort. Randomized control trials (RCTs) and double-blind experiments have typically been performed, but these previous studies were limited by including only a small number of study subjects. The number of subjects ranged from 37^15^ to 326^20^, which may not be enough to explain the difference in efficacy of antipyretics. The studies also only compared antipyretic effects after a set time interval, which varied from 30 minutes to 8 hours. In addition, these studies were conducted in hospital settings, although antipyretics are not only administered in hospitals.

The shortcomings of these previous studies make them lack the power to effectively explain differences in antipyretic effects. Therefore, we decided to investigate the differences in temperature-reducing effects of antipyretics according to type based on real-world data of a large huge number of subjects. We used >4 million and >1.6 million user-reported body temperature and antipyretic treatment records, respectively. The large number of records is enough to explain the differential efficacy of antipyretics. In addition, we studied the different trends of the efficacy of antipyretics at various time intervals. Our hypothesis was that different antipyretics (AA, IBU, and DEX) have different temperature-reducing effects.

## Results

Table 1 shows the descriptive statistics for the weight, antipyretic dose, sex, and age of children with a record of antipyretic treatment. The mean weight and age of the children were significantly different among the antipyretic drug types. This expectedly reflected the relative abundance of IBU and DEX prescriptions in older children and that of acetaminophen in younger children.

**Table 1:** General characteristics of subjects.

Variable	Total (n = 217962)		Subgroup by type of antipyretics						
			AA (n = 82133)		IBU (n = 60770)		DEX (n = 75059)		
	Mean	Standard deviation	Mean	Standard deviation	Mean	Standard deviation	Mean	Standard deviation	*P*-value^a^
Weight (kg)	12.8	3.5	12.5	3.4	13.3	3.8	12.8	3.3	<0.001
Intake (mg/kg)	8.3	3.1	11.9	1.3	7.1	0.9	5.2	0.6	<0.001
Ratio of male sex	0.50		0.50		0.49		0.50		0.24
Age (days)	740.4	501.7	694.6	482.6	782.4	540.4	756.5	485.4	<0.001

^a^Student’s *t*-test used to analyze different distributions of each categorical variable by antipyretics group.

We conducted a one-way analysis of variance (ANOVA) to determine whether there were differences in body temperature between the groups administered AA, IBU, and DEX at each time interval. The ANOVA showed that the difference between the 0–1 hour and 5–6 hour intervals were statistically significant, and we further analyzed the difference between groups using the Games-Howell test. The results are provided in supplemental Tables 1 and 2. Except for the results of the 0–1-hour time interval in the AA and IBU group, all differences were statistically significant (*P* < 0.001). At the 1–2-, 2–3-, and 3–4-hour time intervals, the average body temperatures of the IBU and DEX groups differed from that of the AA group by approximately 0.2 °C, while other differences were ≤0.10 °C.

**Table 2:** Factors associated with average body temperature at specific time intervals analyzed using multiple linear regression.

Variable	Average body temperature at 0–1 hour			Average body temperature at 1–2 hour			Average body temperature at 2–3 hour		
	β	*P*	*95% CI*	β	*P*	*95% CI*	β	*P*	*95% CI*
Intercept									
	38.76	<0.001	38.75–38.77	38.10	<0.001	38.08–38.11	37.98	<0.001	37.96–38.01
Antipyretics									
AA									
IBU	0.00	0.132	−0.01–0.00	−0.18	<0.001	−0.19–0.17	−0.25	<0.001	−0.26 to −0.24
DEX	0.02	<0.001	0.01–0.02	−0.24	<0.001	−0.25–0.24	−0.28	<0.001	−0.29 to −0.27
Weight (kg)									
	0.00	0.906	−0.00–0.00	0.00	<0.001	−0.01 to −0.00	−0.01	<0.001	−0.01 to −0.01
Age (year)									
	0.00	<0.001	−0.00 to −0.00	0.00	<0.001	0.00–0.00	0.00	<0.001	0.00–0.00
Sex									
	0.01	<0.001	0.01–0.01	0.02	<0.001	0.02–0.03	0.02	<0.001	0.02–0.03
**Variable**	**Average body temperature at 3–4 hour**			**Average body temperature at 4–5 hour**			**Average body temperature at 5–6 hour**		
	**β**	***P-*value**	***95% CI***	**β**	***P-*value**	***95% CI***	**β**	***P-*value**	***95% CI***
Intercept									
	38.12	<0.001	38.09–38.15	38.19	<0.001	38.16–38.23	38.23	<0.001	38.20–38.27
Antipyretics									
AA									
IBU	−0.18	<0.001	−0.20 to −0.17	−0.03	<0.001	−0.04 to −0.02	0.06	<0.001	0.04–0.07
DEX	−0.12	<0.001	−0.13 to −0.10	0.07	<0.001	0.06–0.09	0.12	<0.001	0.11–0.13
Weight									
	−0.01	<0.001	−0.01 to −0.01	−0.01	0.003	−0.01 to −0.00	−0.01	0.001	−0.01 to −0.00
Age									
	0.00	0.208	−0.00–0.00	0.00	0.021	−0.00 to −0.00	0.00	0.636	−0.00–0.00
Sex									
	0.00	0.522	−0.01–0.01	−0.01	0.255	−0.02–0.00	0.00	0.924	−0.01–0.01

AA: Acetaminophen, IBU: Ibuprofen, DEX: Dexibuprofen, CI: Confidence Interval Unit: °C.

Table 2 shows the effects of antipyretic type, weight, age, and sex on average body temperature over time according to a multivariate linear regression model and the 95% confidence intervals. The IBU group had 0.18 °C, 0.24 °C, and 0.18 °C (all *P* < 0.001) lower body temperatures than the AA group did at the 1–2, 2–3, and 3–4 hour intervals, respectively, controlling for weight, sex, and age. The DEX group had 0.24 °C (*P* < 0.001), 0.28 °C (*P* < 0.001), and 0.12 °C (*P* < 0.001) lower body temperature than the AA group at the 1–2-, 2–3-, and 3–4-hour intervals, respectively, controlling weight, sex, and age. The IBU and DEX groups showed lower body temperatures than the AA group, which was weakened at the 4–5-hour interval and reversed at the 5–6-hour interval, as shown in Table 1.

**Figure 1:**
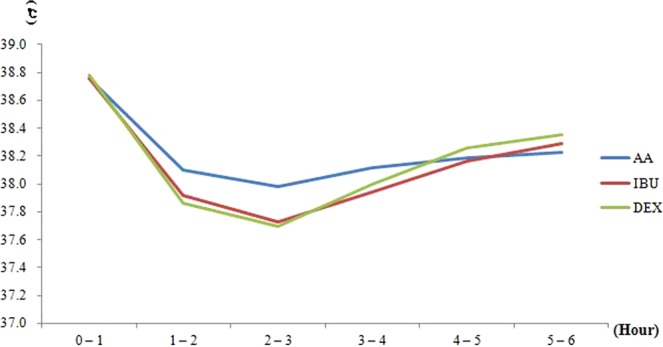
Change in average body temperatures over time, according to antipyretic type.

Using the intercept and the coefficients of each antipyretic series from the multivariate linear model, we calculated the average body temperature after excluding the influence of age, sex, and weight (Table 3, Fig. 1). As shown in Table 2, IBU and DEX reduced body temperature by >1.0 °C at the 2–3-hour interval compared with the average body temperature at the 0–1-hour interval.

**Table 3:** Calculated average body temperatures after excluding effects of age, sex, and weight.

Time interval after antipyretic administration	0–1 hour	1–2 hour	2–3 hours	3–4 hour	4–5 hour	5–6 hour
**Antipyretics**						
AA	38.76	38.10	37.98	38.12	38.19	38.23
IBU	38.76	37.92	37.73	37.94	38.16	38.29
DEX	38.78	37.86	37.70	38.00	38.26	38.35

AA: Acetaminophen, IBU: Ibuprofen, DEX: Dexibuprofen, Unit: °C.

To further analyze the varying efficacy of antipyretics in reducing body temperature, subgroup analyses were performed. Supplementary Table 3 presents the coefficients of linear regression of IBU and DEX in different age subgroups. The 1–5 years old age group showed higher temperature reduction than that of the <1 year old group.

Supplementary Table 4 presents the coefficients of linear regression of IBU and DEX with different initial body temperatures. A similar decrease in temperature and recovery trend after antipyretic administration was observed in the different subgroups, whereas the body temperatures varied. In particular, in children with body temperature ≥40.0 °C, the antipyretics reduced the body temperature by approximately 1.5 °C for AA and 0.2 °C more for IBU and DEX.

## Discussion

This study compares the effectiveness of antipyretics in reducing the body temperature of children based on the data collected using a mobile healthcare application, FeverCoach. The data were filtered and categorized into three treatment groups: AA, IBU, and DEX. Body temperature records were grouped according to the time after administration of the antipyretics. Mean body temperatures were calculated to examine general trends in the temperature-reducing effects. A one-way ANOVA and post-hoc analysis were used to calculate differences in body temperatures among the groups and Multivariate linear regression was used to compare the temperature-reducing effect of IBU and DEX treatment with that of AA, controlling for sex, age, and weight. Our analysis showed that IBU and DEX exhibited better antipyretic effects than AA did, at time intervals of 1–4 hours.

Figure 1 shows that the antipyretics clearly reduced body temperature, which was maximal at the 2–3-hour interval. Additionally, the average body temperature started to rise above 38.0 °C at the 4–5-hour interval. These results are consistent with those other studies of the effective duration of antipyretic action^9^, corroborating the validity of the FeverCoach database. Based on our analysis, we suggest a guideline to recommend alternating antipyretics and encouraging parents to observe and measure the body temperature of children for at least approximately 2 hours. This is because some parents are often anxious when their child has a fever, leading to excessive administration of antipyretics.

The results of the comparison between AA and IBU demonstrated that from the 1–2-hour interval to the 3–4-hour interval, IBU treatment reduced body temperature by approximately 0.2 °C more than AA treatment did, controlling for the influence of weight, age, and sex. Previous studies also compared the antipyretic efficacy of AA and IBU, but the results are not in consensus^15,16,20–22^. While one study found no significant differences between the effects of AA and IBU^21^, a few other studies suggested that IBU offers better antipyretic effects than those of AA^16,20,22^. A meta-analysis reported that IBU may have better antipyretic effects than AA^16^. Our results support previous findings that IBU administered at the proper dose was more effective at reducing body temperature than AA was^16,20,22^.

The results of the comparison between AA and DEX were similar to those of the comparison between AA and IBU. The DEX group had an approximately 0.2 °C lower average body temperature than the AA group did in the 1–4-hour interval. These results show that DEX reduced body temperature better than AA did, when properly prescribed. The antipyretic effect of DEX has been previously verified^19^, but no studies have compared it with those of AA. To the best of our knowledge, this is the first work to compare the temperature-reducing effects of AA and DEX. We suggest that since DEX and IBU have nearly the same reaction pathway, their temperature-reducing effects area also similar.

The superior antipyretic efficacy of IBU and DEX over AA reversed from the 4–5-hour time interval to the 5–6-hour time interval. The average body temperatures of the IBU and DEX groups were 0.06 °C and 0.12 °C higher, respectively than that of the AA group was at the 5–6-hour time interval, which is considerable. This may suggest that the onset of the temperature-reducing effect of IBU and DEX is shorter than that of AA. Further studies should be conducted to compare the onset time of the temperature-reducing effects of different antipyretics.

The comparison between IBU and DEX showed they exhibited similar effects on reducing body temperature. The body temperature difference at each time interval between IBU and DEX did not exceed 0.1 °C. Therefore, we concluded that IBU and DEX are not very different in terms of antipyretic effect. This result is also consistent with that of a previous study, which found that DEX had a similar temperature-reducing effect to that of IBU^19^.

The overall comparison shows the similar antipyretic effect of IBU and DEX, which is better than that of AA in 1–4-hour time interval. This analysis may provide evidence to support the clinical use of IBU and DEX rather than AA in diseases where body temperature management is important such as febrile convulsion and neurologic diseases including Moyamoya disease. The exception would be where there is a specific contraindication for administering IBU and DEX.

Subgroup analysis showed that for children aged 1 to 5, the observed decreases in body temperature after antipyretic administration were greater, and the difference between AA and IBU or DEX were also larger at time intervals of 1–4 hours than at other intervals. However, since the initial body temperature, which influences the quantitative reduction was high, further analysis should be conducted to determine why initial body temperatures of the 1 to 5 years old subgroup were higher.

Subgroup analysis also showed that children with high fever (≥40 °C) exhibited a dramatic reduction in body temperature. However, the reduction may not be solely due to drug efficacy because a variety of cooling methods could have been used by parents according to the app’s guide. However, the efficacy difference observed between AA and IBU or DEX was consistent in this subgroup, which is noteworthy.

This study has some limitations associated with the nature of the data used. First, the data were recorded by parents who are usually not professional health care providers. Therefore, the method and position used for temperature measurement and thermometer type may have differed among patients. Furthermore, parents may have made other efforts to decrease the children’s body temperature, especially because the application provides a detailed guide for this. In addition, in some cases, the accurate body temperature may not have been recorded. Another weakness is that while we did not exclude the records of children taking antibiotics because the data were unavailable, antibiotics could also affect children’s fever. We did not include neonates and children <6 months old or those who alternated antipyretics, so further study would be needed to analyze the efficacy of alternating antipyretics.

Despite these limitations, this study also has several strengths compared with previous studies. For instance, our results are based on an incomparably larger self-reported dataset than those used in previous studies. The small sample sizes of previous studies may have decreased their explanatory power. A large self-reported dataset not only has excellent explanatory power when properly filtered, but also better mimics a real-world setting. Furthermore, to the best of our knowledge, this study is the first to compare AA, IBU, and DEX. Although previous studies have compared the temperature-reducing efficacy of two types of antipyretics, most focused on comparing AA and IBU. To the best of our knowledge, this is the first study to compare the antipyretic effect between an AA-treated and DEX-treated group.

In conclusion, IBU and DEX were more effective in reducing body temperature than AA was and they showed no apparent differences in clinical efficacy of their antipyretic effects, which suggests they likely have similar mechanisms of body temperature reduction. The average cost (US dollar/kg) of DEX is 3.7 times more than that of IBU^23,24^. The choice of antipyretics for children may be influenced by the underlying medical condition, chronic medications, adverse effects, and cost-benefit ratio. Consequently, the decision on the type of antipyretic to use in children should be made on a case-by-case basis.

## Methods

### Mobile healthcare application, fevercoach

FeverCoach is a mobile healthcare application targeted at parents with children exhibiting various symptoms, including fever. The application provides parents with guidelines to assess a child’s condition and is especially useful for parents who cannot go to the hospital. This application allows parents to effectively and accurately control fever symptoms. The application mainly offers the following functions: data storage for each child’s body temperature, guidance on antipyretic drug administration, and general pediatric health information. Detailed explanations of the application and its data storage method and screenshots of the application are provided in Appendix 1.

The application was released more than 2 years ago, and it has continued to accumulate data. From July 2015 to June 2017, approximately 4.4 million body temperature and approximately 1.6 million antipyretic treatment records were accumulated. We believe that this dataset could be very valuable for use in a variety of services or studies.

### Data and study population

For this study, we mainly extracted two types of data from the FeverCoach database: body temperature measurement and antipyretic treatment records. To guarantee the validity of the analysis, we filtered records by various conditions.

### Body temperature measurement records

Parents input three values (body temperature, time of body temperature measurement in minutes, and vaccination information) after measuring their child’s body temperature, which was recorded to the first decimal place. Whether the child had a vaccination within the last 48 hours is also input because vaccinations can significantly affect body temperature. Every measurement is stored in the FeverCoach database with time-stamped identifier variables that distinguish each child. There were 4,424,820 body temperature measurements recorded in the dataset.

### Antipyretic treatment records

Parents input five values (administration method, antipyretic type, product name, dosage, and treatment time) after antipyretic administration to their child. Parents had four administration methods to choose from (potions, powder, pills, and suppositories) and three antipyretic types (AA, IBU, and DEX). Parents also had the option to input the drug’s product name. Treatment doses were entered in milliliters or milligrams, and treatment time was entered in minutes. Similarly, every treatment record was stored with identifier variables that distinguish each child and a timestamp. There were 1,630,707 antipyretics treatment records in the dataset.

### Study population

**Figure 2:**
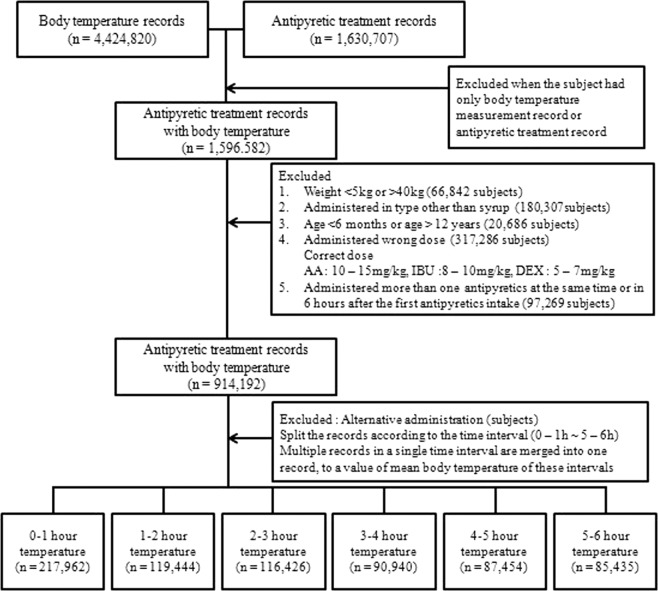
Inclusion and Exclusion flow chart.

Because our results were derived from self-reported data, it was especially crucial to filter out inadequate records in the database. Our inclusion and exclusion flowchart is provided in Fig. 2. We excluded records incomplete age or weight data. Children >12 years old or <6 months and those weighing >40 kg or <5 kg were considered to potentially undermine the validity of our analyses and were excluded. Six months was chosen as the lower age cutoff because children younger than 6 months are prohibited from being prescribed IBU and DEX. The upper age cutoff of 12 years old was chosen because children >12 years old are administered the adult dose. At least 99% of the 6-month-old children weighed at least 5 kg, and the top 50% of 12-year-old children weighed at least 40 kg.

Furthermore, invalid antipyretic administration was excluded from our analysis. All body temperature measurements not recorded within 6 hours after antipyretic treatment were considered inadequate and, therefore, were excluded. Additionally, we excluded any records of antipyretics not administered orally and those where patients received additional treatment within 6 hours. We focused on orally administered drugs because children rarely take IBU and DEX via other routes. Records with more than one antipyretic treatment in 6 hours (alternating antipyretic intakes or records of same drug administration) were also excluded. Specifically, among 97,269 records excluded because of more than one administration of antipyretics in 6 hours, 35,926 were administered AA twice, 23,128 IBU twice, 29,047 DEX twice, 3,468 AA and IBU, 3,007 IBU and DEX, 2,590 DEX and AA, and 103 were administered all three agents (AA, IBU and DEX). Finally, we also excluded data of body temperatures taken within 5 minutes of administering antipyretic treatment and those that were lower than 38.0 °C, because antipyretics are only supposed to be administered when the patient is feverish.

After our exclusions, 217,962 of the 1,630,707 antipyretic treatment records were analyzed, consisting of 82,133, 60,770, and 75,059 AA, IBU, and DEX treatment records, respectively.

### Ethical considerations

All data were anonymized at the time of storage. In addition, the Yonsei University Health System Institutional Review Board (IRB), Seoul, Korea provided formal ethics approval of this study (IRB approval number 4-2017-1074) that waived the need for informed consent as part of the study approval. Consequently, the data analysis was performed without requiring consent from the participants.

### Variables

The main independent variable for our analysis was antipyretic type, which was categorized into three groups: AA, IBU, and DEX. The age, weight, and sex of a child are well known to influence temperature-reducing effects and, therefore were also included in the analysis.

We included body temperature as a dependent variable with six time intervals: 0–1, 1–2, 2–3, 3–4, 4–5, and 5–6 hours. Previous studies fixed the time interval between antipyretic treatment and body temperature measurements. However, as our data was user-recorded, the time intervals between antipyretic treatment and body temperature measurements were random. Therefore, we divided the body temperature measurements after antipyretic treatment by 1-hour intervals. For a single antipyretic treatment record, if multiple body temperature measurements were recorded in the same 1-hour interval, the average of those records was calculated for that time interval. For a single antipyretic treatment record, if no records existed in a single time interval, the data field for that time interval was left empty. Therefore, only records with at least one observation in a 1-hour time interval were included in the analysis of that time interval.

### Statistical analysis

We first examined the general characteristics of antipyretic records. The Student *t*-test was performed to analyze differences in populations between antipyretic drug groups. Averages and standard deviations of body temperatures by time interval were calculated for the data on the three groups of antipyretics (AA, IBU, DEX). A one-way ANOVA and post-hoc test were performed to compare average body temperatures of the three antipyretic groups. Because the data size was large enough, we choose ANOVA for the analysis, which also enabled the calculation of the effect size in degrees Celsius, which is easy to interpret for both researchers and clinicians. Temperature changes over time were analyzed as categorical variables. Next, we performed multivariate linear regression analysis to investigate the relationships between antipyretic type and other independent variables, after stratifying body temperature by time interval. Dose was not included in the model since the optimal dose differed by antipyretic type, making a pair-wise comparison between two drugs difficult. Instead, only records that adhered to the optimal dose were included in the analysis. Finally, data were subgrouped by age and initial body temperature. Effect sizes for variables were also calculated using the coefficients of linear regression. The AA group was used as the reference in our models. We determined that P-values <0.05 indicated statistical significance. All statistical analyses were performed using R statistical software (Foundation for Statistical Computing, Vienna, Austria), a free software program for statistical computation and graphical presentation.

## Supplementary information

Supplementary information
