# Publisher Correction: Comparison of Fever-reducing Effects in Self-reported Data from the Mobile App: Antipyretic Drugs in Pediatric Patients

**DOI:** 10.1038/s41598-020-64762-2

**Published:** 2020-05-12

**Authors:** Jiyun Choi, Seyun Chang, Jong Gyun Ahn

**Affiliations:** 1 Yonsei University College of Medicine, Seoul, Republic of Korea; 2 Pohang University of Science and Technology, Pohang, Republic of Korea; 3 Mobile Doctor Co., Ltd, Seoul, Republic of Korea; 4 Department of Pediatrics, Severance Children’s Hospital, Yonsei University College of Medicine, Seoul, Republic of Korea

Correction to: *Scientific Reports* 10.1038/s41598-020-60193-1, published online 03 March 2020

In the original version of this Article, the author Jong Gyun Ahn was incorrectly indexed. This error has now been corrected.

