# Supplementary material for: Comparison of Fever-reducing Effects in Self-reported Data from the Mobile App: Antipyretic Drugs in Pediatric Patients
Source: Sci Rep. 2020 Mar 3;10:3879. doi: 10.1038/s41598-020-60193-1 (PMC7054323; doi:10.1038/s41598-020-60193-1)
Supplement: Supplementary file 1 — Supplementary information [file 41598_2020_60193_MOESM1_ESM.docx]

**Comparison of Fever-reducing Effects in Self-reported Data from the Mobile App: Antipyretic Drugs in Pediatric Patients**

*Jiyun Choi^1,3¶^, Seyun Chang^2,3&^, and Jong Gyun Ahn^4*^*

^1^ Yonsei University College of Medicine, Seoul, Republic of Korea

^2^ Pohang University of Science and Technology, Pohang, Republic of Korea

^3^ Mobile Doctor Co., Ltd., Seoul, Republic of Korea

^4^ Department of Pediatrics, Severance Children’s Hospital, Yonsei University College of Medicine, Seoul, Republic of Korea

^¶^ First author

^&^Second author

*** Corresponding author**

**Address correspondence to:** Department of Pediatrics, Severance Children’s Hospital, Yonsei University College of Medicine, 50-1, Yonsei-ro, Seodaemun-gu, Seoul, 03722

E-mail: jgahn@yuhs.ac

Tel: +82-2-2228-2057

Fax: +82-2-393-9118

**S1. Appendix**

**Pediatric fever managing mobile app “Fevercoach” and its data processing**

**Introduction of the application** “Fevercoach” is a healthcare mobile application that targets parents with children showing various symptoms, including fever. The application provides parents with a guideline regarding the condition of the child, especially for parents who cannot immediately go to the hospital. This application allows parents to effectively and accurately control fever symptoms. The application mainly serves the following functions: saving records of children's body temperatures, suggesting guidelines for antipyretics intake, and providing pediatric health information. Detailed explanation of the application is provided in supplementary material.

The main service of ‘Fevercoach’ application is to provide appropriate information related to feverish situations. When parents input their children’s temperatures, the application shows guidelines using status illustration (having fever or not), antipyretic intake, and therapies. If the child has a fever, the application can be set to spontaneously give alarms to check the child’s temperature again in every 30 minutes. The alarm will continue until the situation ends, meaning that the child’s temperature has reached the normal range. Once the situation ends, parents can check records of previous body temperatures and guideline histories using the app’s timeline function.

“Fevercoach” also provides antipyretic treatment guidelines. The application guides parents to give the appropriate volume of antipyretic medicine based on the children’s weight, and not to give exceed the maximum daily dose. After giving the antipyretics, parents are suggested to watch over their children's state for two hours, and then take additional actions, such as measuring the children’s body temperature again or giving them antipyretic.

The application provides more specific guidelines according to children's diagnosis and symptoms. Parents can input names of diagnosis and symptoms. The application also offers an additional coping method using its own algorithm for different diagnosis and symptoms. For example, information on what to do with antipyretics is provided as an additional message depending on children's diagnosis and symptoms.

Lastly, the application also gives useful information for pediatric health, such as details on children’s healthcare, pandemic alerts, and fine dust concentration alerts. In particular, information on children's healthcare includes proper methods for antipyretics intake, illustration of fever-related diseases, and fever management tips. Screenshots of the application are provided in Figure A, Figure B, Figure C, Figure D, and Figure E.


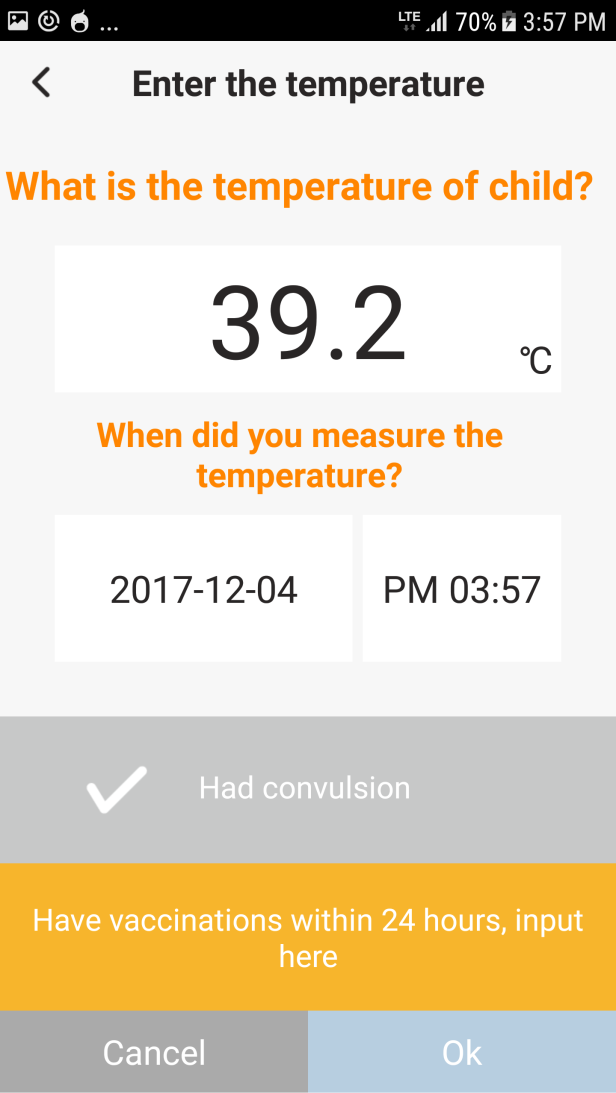

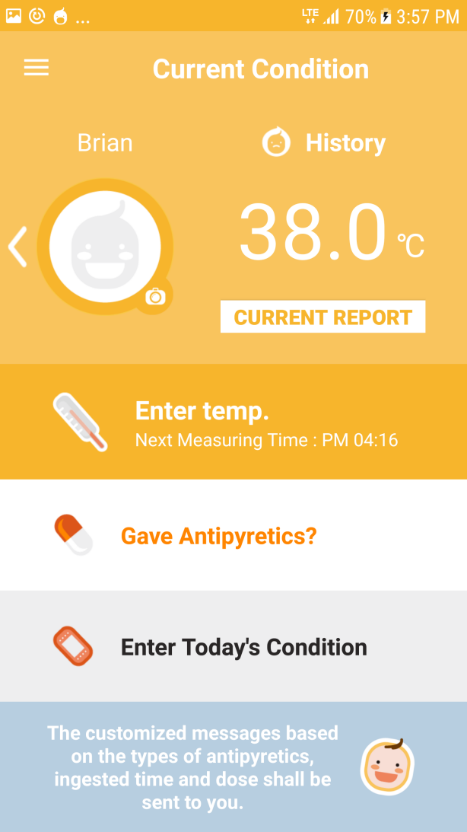


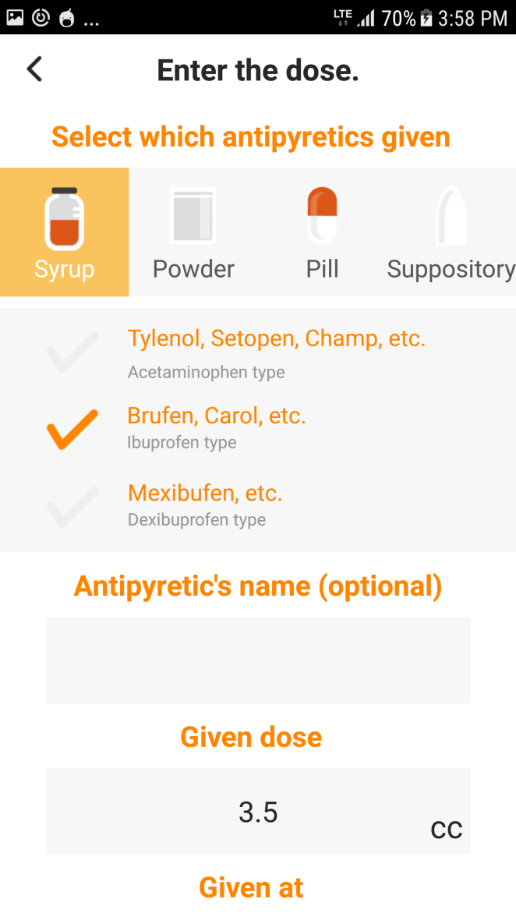

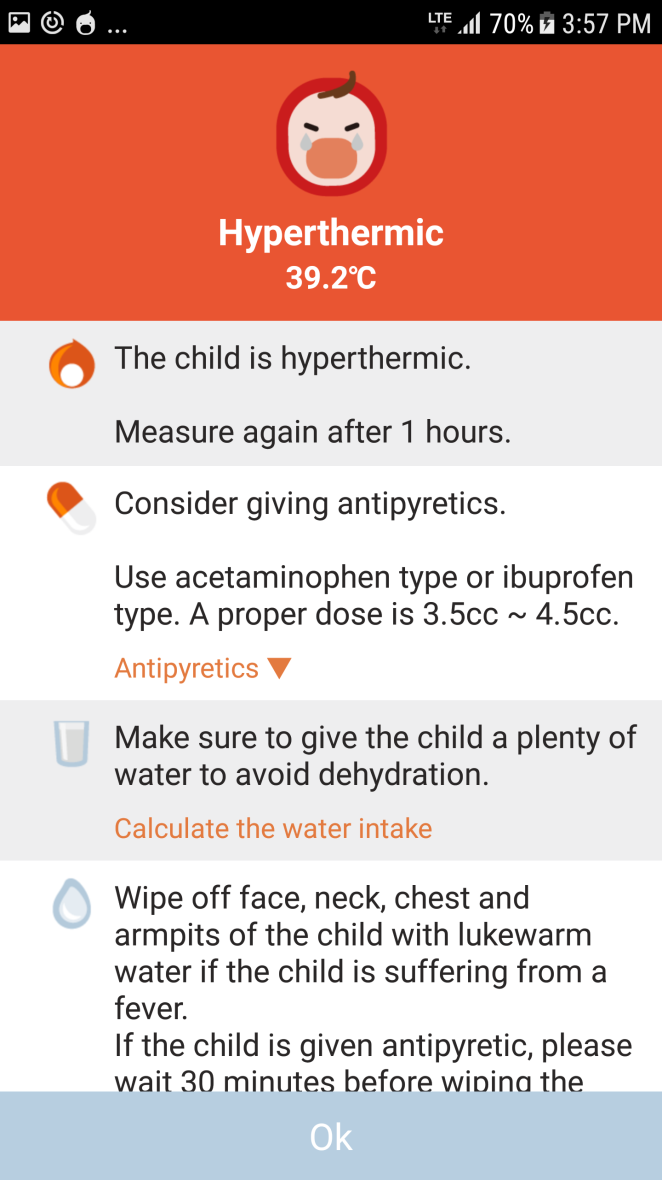


Figure D. Antipyretics treatment input page

Figure C. Report page

(with information on fever management)

Figure B. Temperature input page

Figure A. Main Page


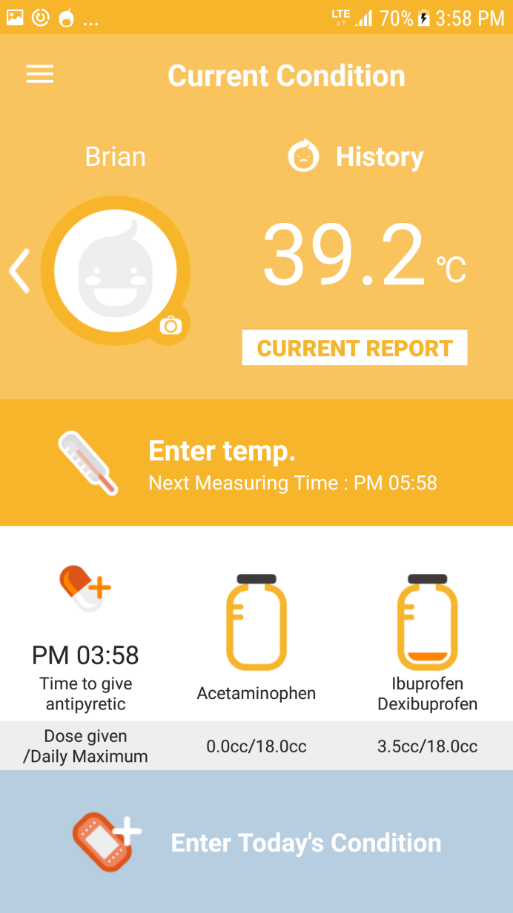


Figure E. Main page (after
input antipyretics treatment)

**Data types collected by application** Types of data collected at the application database are listed below. In general, there are two types of data: data that are automatically generated data, and data that are input by parents. Especially, for the data that were input by parents, two family medicine experts chose the proper wordings that are familiar for most parents. These records have no medical or legal effects.

- 1. Device ID
     - This data is automatically generated when a parent downloads the app. It indicates ID of the installed device.
  2. Baby information

- This data is input by parents when they register their children’s information into the app. Parents must input their children's nickname, weight, sex, and birthday.

- 1. Baby number

- This data is automatically generated when parents register their children in the app. It indicates the order of registered children in single device. Baby number starts from 1, and increases by 1 when a new child gets registered to the same device.

- 1. Body temperature

- This data is input by parents. The digits can be entered up to one decimal point, according to the reading on thermometer when parents measured their children's body temperature.

- 1. Body temperature measurement time

- This data is automatically generated when parents input body temperature. Once body temperature is recorded, the body temperature measurement time (UTC) is automatically generated within the app.

- 1. Antipyretic treatment

- This data is input by parents. It includes information on formulation (potion, powder, pill, suppository), series (acetaminophen series, ibuprofen series, dexibuprofen series), product name, dose of treatment (in mL or mg, depending on formulation), and the time of treatment.

- 1. Symptoms

- This data is input by parents, who can choose multiple symptoms of their children from the following list: cough, runny nose, phlegm, wheeze, fast breathing/panting, barking cough, sneeze, throat-ache, sag seriously, whines much, eat less than 80%, eat less than 60%, eat less than 40%, urine decreased, vomit, diarrhea, constipation, stomachache, body rash, bubbles at hands/feet, bubbles in mouth, headache, jaundice, BCG flare, congestion, raspberry tongue, and seizure.

- 1. Diagnosis

- This data is input by parents. After getting a proper diagnosis of their children’s disease from a doctor, parents can choose a single diagnosis from this list: bronchitis, bronchiolitis, pneumonia, adenovirus infection, laryngitis, pharyngitis/tonsilitis, common cold, influenza, canker sore, herpangina, hand-foot-and-mouth disease, enteritis, menigitis, otitis media, UTI, mumps, scarlet fever, herpes zoster, and measles.

- 1. Location information

- This data is automatically generated when parents input body temperature, antipyretic treatment, symptoms, and diagnosis. Latitude and longitude values are shown only for users who consented to provide their location information.

- 1. Vaccination information

- This data is input by parents when they enter children’s body temperature. Parents may check the item that says "have vaccinations within 24 hours, input here" when they enter the body temperature of their children. Given that their children received vaccinations within 24 hours before measuring their body temperature, parents can choose multiple vaccination types from this list: BCG, Hepatatis B, DTaP, MMR, Japanese encephalitis, pneumococcemia, Hib, polio, rotavirus, hapariris A, Chickenpox, and influenza.

- 1. User memo

- This data is input by parents when they have something to note.

**Methods of storing and de-identifying user information** By parents’ input, data on children’s body temperature, antipyretic treatment, symptoms, or diagnosis is stored in the Fevercoach database as a log data, which includes baby information. For example, when parents enter the body temperature information, device ID, baby information, baby ID, body temperature, body temperature measurement time, vaccination information, and location information (optional) are all stored in a single row.

The application doesn't store individual names. Despite personal information that are stored in the server (i.e., date of birth, weight, sex, and etc.), even if these information are combined. Information on specific identity of individuals (i.e., name and resident registration number) cannot be saved in the app. Therefore, every data is de-identified.

**Data availability**

The raw FeverCoach data is uploaded at Synapse:

https://www.synapse.org/#!Synapse:syn21438141/tables/ .

**Supplementary tables**

| **Supplementary Table 1. One-way ANOVA showing comparison about average body temperature in certain time intervals between and within groups** | | | | | | | | | | | | | |
| --- | --- | --- | --- | --- | --- | --- | --- | --- | --- | --- | --- | --- | --- |
|  | Average body temperature at 0-1 hour | | | | |  | Average body temperature at 1-2 hour | | | | | | |
|  | Sum of  Squares | Degree of  Freedom | Mean Square | *F* | *P*^a^ |  | Sum of  Squares | Degree of  Freedom | Mean Square | | *F* | | *P* |
| Between groups | 20.65 | 2 | 10.33 | 38.62 | <.001 |  | 1349.43 | 2 | 674.72 | | 1726.83 | | <.001 |
| Within groups | 58268.30 | 217959 | 0.27 |  |  |  | 46668.77 | 119441 | 0.39 | |  | |  |
| Total | 58288.95 | 217961 |  |  |  |  | 48018.20 | 119443 |  | |  | |  |
|  |  |  |  |  |  |  |  |  |  | |  | |  |
|  | Average body temperature at 2-3 hour | | | | |  | Average body temperature at 3-4 hour | | | | | | |
|  | Sum of  Squares | Degree of  Freedom | Mean Square | *F* | *P* |  | Sum of  Squares | Degree of  Freedom | Mean Square | *F* | | *P* | |
| Between groups | 1992.09 | 2 | 996.04 | 1902.19 | <.001 |  | 569.11 | 2 | 284.55 | 389.34 | | <.001 | |
| Within groups | 60962.78 | 116423 | 0.52 |  |  |  | 66462.62 | 90937 | 0.73 |  | |  | |
| Total | 62954.87 | 116425 |  |  |  |  | 67031.73 | 90939 |  |  | |  | |
|  |  |  |  |  |  |  |  |  |  |  | |  | |
|  | Average body temperature at 4-5 hour | | | | |  | Average body temperature at 5-6 hour | | | | | | |
|  | Sum of  Squares | Degree of  Freedom | Mean Square | *F* | *P* |  | Sum of  Squares | Degree of  Freedom | Mean Square | *F* | | *P* | |
| Between groups | 158.82 | 2 | 79.41 | 101.34 | <.001 |  | 223.221 | 2 | 111.61 | 160.06 | | <.001 | |
| Within groups | 68524.31 | 87451 | 0.78 |  |  |  | 59572.132 | 85432 | 0.70 |  | |  | |
| Total | 68683.13 | 87453 |  |  |  |  | 59795.35 | 85434 |  |  | |  | |
|  |  |  |  |  |  |  |  |  |  |  | |  | |
| a. 3 groups were compared, each took AA, IBU, DEX respectively | | | | | | | | | | | | | |

| **Supplementary Table** 2. Multiple comparison of average body temperature at certain time intervals | | | | | | | |
| --- | --- | --- | --- | --- | --- | --- | --- |
| Time interval | Group1 | Group2 | Mean difference (Group1 - Group2) | SE | *P*^a^ | 95% CI | |
|  |  |  |  |  |  | *Lower bound* | *Upper bound* |
| 0-1 hour | AA | IBU | -0.01 | 0.00 | .11 | -0.01 | 0.00 |
|  | AA | DEX | 0.02 | 0.00 | <.001 | 0.01 | 0.02 |
|  | IBU | DEX | 0.02 | 0.00 | <.001 | 0.02 | 0.03 |
| 1-2 hour | AA | IBU | -0.18 | 0.00 | <.001 | -0.19 | -0.17 |
|  | AA | DEX | -0.24 | 0.00 | <.001 | -0.25 | -0.23 |
|  | IBU | DEX | -0.06 | 0.00 | <.001 | -0.07 | -0.05 |
| 2-3 hour | AA | IBU | -0.26 | 0.00 | <.001 | -0.27 | -0.24 |
|  | AA | DEX | -0.28 | 0.00 | <.001 | -0.29 | -0.27 |
|  | IBU | DEX | -0.02 | 0.00 | <.001 | -0.03 | -0.01 |
| 3-4 hour | AA | IBU | -0.19 | 0.01 | <.001 | -0.21 | -0.18 |
|  | AA | DEX | -0.12 | 0.01 | <.001 | -0.14 | -0.10 |
|  | IBU | DEX | 0.07 | 0.01 | <.001 | 0.05 | 0.09 |
| 4-5 hour | AA | IBU | -0.04 | 0.01 | <.001 | -0.06 | -0.02 |
|  | AA | DEX | 0.07 | 0.01 | <.001 | 0.05 | 0.09 |
|  | IBU | DEX | 0.11 | 0.01 | <.001 | 0.09 | 0.13 |
| 5-6 hour | AA | IBU | 0.05 | 0.01 | <.001 | 0.03 | 0.07 |
|  | AA | DEX | 0.12 | 0.01 | <.001 | 0.10 | 0.14 |
|  | IBU | DEX | 0.07 | 0.01 | <.001 | 0.05 | 0.09 |
| a. Result of Games - Howell test was performed as a post-hoc analysis. | | | | | | | |

| **Supplementary Table 3. Coefficients of AA, IBU, DEX in linear regression body temperature in age subgroups** | | | | | | | | | | | |
| --- | --- | --- | --- | --- | --- | --- | --- | --- | --- | --- | --- |
| **Variable** | **Average body temperature  at 0-1hour** | | |  | **Average body temperature  at 1-2hour** | | |  | **Average body temperature  at 2-3hour** | | |
|  | **β** | ***p*-value** | ***95% CI*** |  | **β** | ***p*-value** | ***95% CI*** |  | **β** | ***p*-value** | ***95% CI*** |
| **Age ≤ 1 year** |  |  |  |  |  |  |  |  |  |  |  |
| **Intercept** | 38.41 |  | 38.36 - 38.46 |  | 37.73 |  | 37.65 - 37.82 |  | 37.53 |  | 37.43 - 37.63 |
| **Antipyretics** |  |  |  |  |  |  |  |  |  |  |  |
| AA |  |  |  |  |  |  |  |  |  |  |  |
| IBU | 0.02 | 0.003 | 0.01 - 0.04 |  | -0.09 | <0.001 | -0.11 - -0.06 |  | -0.14 | <0.001 | -0.17 - -0.11 |
| DEX | 0.06 | <0.001 | 0.04 - 0.07 |  | -0.14 | <0.001 | -0.16 - -0.11 |  | -0.14 | <0.001 | -0.17 - -0.11 |
| **1 year < Age ≤ 5 years** |  |  |  |  |  |  |  |  |  |  |  |
| **Intercept** | 38.79 |  | 38.78 - 38.81 |  | 38.00 |  | 37.98 - 38.03 |  | 37.96 |  | 37.93 - 37.98 |
| **Antipyretics** |  |  |  |  |  |  |  |  |  |  |  |
| AA |  |  |  |  |  |  |  |  |  |  |  |
| IBU | -0.01 | 0.015 | -0.01 - -0.01 |  | -0.18 | <0.001 | -0.19 - -0.17 |  | -0.25 | <0.001 | -0.26 - -0.24 |
| DEX | 0.01 | 0.002 | 0.01 - 0.02 |  | -0.24 | <0.001 | -0.25 - -0.23 |  | -0.27 | <0.001 | -0.28 - -0.26 |
| **5 years ≤ Age ≤ 12 years** |  |  |  |  |  |  |  |  |  |  |  |
| **Intercept** | 38.70 |  | 38.65 - 38.74 |  | 38.18 |  | 38.10 - 38.26 |  | 37.89 |  | 37.81 - 37.98 |
| **Antipyretics** |  |  |  |  |  |  |  |  |  |  |  |
| AA |  |  |  |  |  |  |  |  |  |  |  |
| IBU | -0.01 | 0.220 | -0.04 - 0.01 |  | -0.22 | <0.001 | -0.26 - -0.18 |  | -0.28 | <0.001 | -0.32 - -0.24 |
| DEX | 0.04 | 0.003 | 0.01 - 0.06 |  | -0.25 | <0.001 | -0.29 - -0.22 |  | -0.30 | <0.001 | -0.35 - -0.26 |
| **Variable** | **Average body temperature  at 3-4hour** | | |  | **Average body temperature  at 4-5hour** | | |  | **Average body temperature  at 5-6hour** | | |
|  | **β** | ***p*-value** | ***95% CI*** |  | **β** | ***p*-value** | ***95% CI*** |  | **β** | ***p*-value** | ***95% CI*** |
| **Age ≤ 1 year** |  |  |  |  |  |  |  |  |  |  |  |
| **Intercept** | 37.61 |  | 37.48 - 37.75 |  | 37.75 |  | 37.60 - 37.90 |  | 37.86 |  | 37.72 - 38.00 |
| **Antipyretics** |  |  |  |  |  |  |  |  |  |  |  |
| AA |  |  |  |  |  |  |  |  |  |  |  |
| IBU | -0.11 | <0.001 | -0.15 - '0.07 |  | -0.03 | 0.145 | -0.08 - 0.01 |  | 0.02 | 0.264 | -0.02 - 0.07 |
| DEX | -0.03 | 0.094 | -0.07 - 0.01 |  | 0.06 | 0.006 | 0.02 - 0.10 |  | 0.13 | <0.001 | 0.09 - 0.17 |
| **1 year < Age ≤ 5 years** |  |  |  |  |  |  |  |  |  |  |  |
| **Intercept** | 38.13 |  | 38.10 - 38.16 |  | 38.25 |  | 38.21 - 38.28 |  | 38.28 |  | 38.24 - 38.31 |
| **Antipyretics** |  |  |  |  |  |  |  |  |  |  |  |
| AA |  |  |  |  |  |  |  |  |  |  |  |
| IBU | -0.19 | <0.001 | -0.20 - -0.17 |  | -0.04 | <0.001 | -0.05 - -0.02 |  | 0.04 | <0.001 | 0.03 - 0.06 |
| DEX | -0.12 | <0.001 | -0.14 - -0.10 |  | 0.05 | <0.001 | 0.04 - 0.07 |  | 0.11 | <0.001 | 0.10 - 0.12 |
| **5 years ≤ Age ≤ 12 years** |  |  |  |  |  |  |  |  |  |  |  |
| **Intercept** | 38.02 |  | 37.90 - 38.13 |  | 38.13 |  | 38.01 - 38.26 |  | 38.20 |  | 38.07 - 38.33 |
| **Antipyretics** |  |  |  |  |  |  |  |  |  |  |  |
| AA |  |  |  |  |  |  |  |  |  |  |  |
| IBU | -0.27 | <0.001 | -0.32 - -0.23 |  | -0.13 | <0.001 | -0.2 - -0.07 |  | 0.01 | 0.863 | -0.05 - 0.07 |
| DEX | -0.17 | <0.001 | -0.23 - -0.12 |  | 0.04 | 0.187 | -0.02 - 0.1 |  | 0.14 | <0.001 | 0.08 - 0.20 |
| AA : Acetaminophen, IBU : Ibuprofen, DEX : Dexibuprofen, CI : Confidence Interval, Unit : ˚C | | | | | | | | | | | |

| **Supplementary Table 4. Coefficients of AA, IBU, DEX in linear regression body temperature in initial temperature subgroups** | | | | | | | | | | | |
| --- | --- | --- | --- | --- | --- | --- | --- | --- | --- | --- | --- |
| **Variable** | **Average body temperature  at 0-1hour** | | |  | **Average body temperature  at 1-2hour** | | |  | **Average body temperature  at 2-3hour** | | |
|  | **β** | ***p*-value** | ***95% CI*** |  | **β** | ***p*-value** | ***95% CI*** |  | **β** | ***p*-value** | ***95% CI*** |
| **Initial temperature<39.0˚C** |  |  |  |  |  |  |  |  |  |  |  |
| **Intercept** | 38.47 |  | 38.46 - 38.48 |  | 37.79 |  | 37.78 - 37.80 |  | 37.68 |  | 37.66 - 37.70 |
| **Antipyretics** |  |  |  |  |  |  |  |  |  |  |  |
| AA |  |  |  |  |  |  |  |  |  |  |  |
| IBU | 0.00 | 0.308 | 0.00 - 0.00 |  | -0.15 | <0.001 | -0.16 - -0.14 |  | -0.21 | <0.001 | -0.22 - -0.20 |
| DEX | 0.01 | <0.001 | 0.01 - 0.01 |  | -0.21 | <0.001 | -0.22 - 0.20 |  | -0.24 | <0.001 | -0.25 - -0.22 |
| **39.0˚C ≤ Initial temperature < 39.9˚C** |  |  |  |  |  |  |  |  |  |  |  |
| **Intercept** | 39.28 |  | 39.28 - 39.28 |  | 38.30 |  | 38.27 - 38.33 |  | 38.20 |  | 38.17 - 38.23 |
| **Antipyretics** |  |  |  |  |  |  |  |  |  |  |  |
| AA |  |  |  |  |  |  |  |  |  |  |  |
| IBU | 0.00 | 0.605 | -0.01 - 0.00 |  | -0.20 | <0.001 | -0.21 - -0.18 |  | -0.27 | <0.001 | -0.29 - -0.25 |
| DEX | 0.00 | 0.877 | 0.00 - 0.01 |  | -0.28 | <0.001 | -0.30 - -0.27 |  | -0.31 | <0.001 | -0.32 - - 0.29 |
| **Initial temperature ≥ 40.0˚C** |  |  |  |  |  |  |  |  |  |  |  |
| **Intercept** | 40.13 |  | 40.11 - 40.16 |  | 38.58 |  | 38.47 - 38.69 |  | 38.63 |  | 38.50 - 38.76 |
| **Antipyretics** |  |  |  |  |  |  |  |  |  |  |  |
| AA |  |  |  |  |  |  |  |  |  |  |  |
| IBU | 0.00 | 0.642 | -0.02 - 0.01 |  | -0.20 | <0.001 | -0.27 - -0.14 |  | -0.29 | <0.001 | -0.37 - -0.21 |
| DEX | -0.01 | 0.397 | -0.02 - 0.01 |  | -0.30 | <0.001 | -0.36 - 0.24 |  | -0.31 | <0.001 | -0.35 - -0.26 |
| **Variable** | **Average body temperature  at 3-4hour** | | |  | **Average body temperature  at 4-5hour** | | |  | **Average body temperature  at 5-6hour** | | |
|  | **β** | ***p*-value** | ***95% CI*** |  | **β** | ***p*-value** | ***95% CI*** |  | **β** | ***p*-value** | ***95% CI*** |
| **Initial temperature<39.0˚C** |  |  |  |  |  |  |  |  |  |  |  |
| **Intercept** | 37.84 |  | 37.82 - 37.87 |  | 38.03 |  | 38.00 - 38.06 |  | 38.10 |  | 38.07 - 38.13 |
| **Antipyretics** |  |  |  |  |  |  |  |  |  |  |  |
| AA |  |  |  |  |  |  |  |  |  |  |  |
| IBU | -0.16 | <0.001 | -0.18 - -0.15 |  | -0.05 | <0.001 | -0.07 - -0.04 |  | 0.02 | 0.076 | 0.00 - 0.03 |
| DEX | -0.10 | <0.001 | -0.12 - -0.08 |  | 0.04 | <0.001 | 0.02 - 0.05 |  | 0.09 | <0.001 | 0.07 - 0.10 |
| **39.0˚C ≤ Initial temperature < 39.9˚C** |  |  |  |  |  |  |  |  |  |  |  |
| **Intercept** | 38.34 |  | 38.30 - 38.38 |  | 38.38 |  | 38.34 - 38.43 |  | 38.39 |  | 38.35 - 38.44 |
| **Antipyretics** |  |  |  |  |  |  |  |  |  |  |  |
| AA |  |  |  |  |  |  |  |  |  |  |  |
| IBU | -0.22 | <0.001 | -0.25 - -0.19 |  | -0.02 | 0.162 | -0.05 - 0.01 |  | 0.08 | <0.001 | 0.06 - 0.11 |
| DEX | -0.14 | <0.001 | -0.17 - -0.12 |  | 0.07 | <0.001 | 0.05 - 0.10 |  | 0.14 | <0.001 | 0.12 - 0.17 |
| **Initial temperature ≥ 40.0˚C** |  |  |  |  |  |  |  |  |  |  |  |
| **Intercept** | 38.67 |  | 38.50 - 38.84 |  | 38.89 |  | 38.71 - 39.07 |  | 38.73 |  | 38.55 - 38.91 |
| **Antipyretics** |  |  |  |  |  |  |  |  |  |  |  |
| AA |  |  |  |  |  |  |  |  |  |  |  |
| IBU | -0.15 | 0.006 | -0.25 - -0.04 |  | 0.00 | 0.997 | -0.11 - 0.11 |  | 0.07 | 0.232 | -0.04 - 0.18 |
| DEX | -0.11 | 0.022 | -0.21 - -0.02 |  | 0.06 | 0.218 | -0.04 - 0.17 |  | 0.17 | 0.001 | 0.07 - 0.27 |
| AA : Acetaminophen, IBU : Ibuprofen, DEX : Dexibuprofen, CI : Confidence Interval, Unit : ˚C | | | | | | | | | | | |
